# Reduction in the IL-6 level at 24 h after admission to the intensive care unit is a survival predictor for Vietnamese patients with sepsis and septic shock: a prospective study

**DOI:** 10.1186/s12873-018-0191-4

**Published:** 2018-11-06

**Authors:** Pham Thi Ngoc Thao, Ton Thanh Tra, Nguyen Truong Son, Koji Wada

**Affiliations:** 1 Cho Ray Hospital, 201B Nguyen Chi Thanh Street, District 5, Ho Chi Minh City, Vietnam; 2 International University of Health and Welfare, Tokyo, Japan

**Keywords:** IL-6, Sepsis, Survival, Intensive care

## Abstract

**Background:**

Sepsis and septic shock are common problems in intensive care units (ICUs). The mortality of patients with sepsis or septic shock is high. We investigated if reduction in the serum concentration of the cytokines tumor necrosis factor α, interleukin (IL)-6 and IL-10, and the rate of change in the IL-6 level at 24 h after ICU admission were survival predictors for patients with sepsis and septic shock in a Vietnamese population.

**Methods:**

This was a prospective study conducted at an ICU in Cho Ray Hospital, Vietnam, from October 2014 to October 2016. Patients diagnosed with sepsis or septic shock using validated international guidelines were enrolled. Plasma samples were collected upon (T_0_) and 24 h after (T_24_) ICU admission for measurement of cytokine concentrations. Blood tests were done to detect organ dysfunction. The duration of ICU stays, hospital stay, APACHE II and SOFA scores, and the in-hospital mortality were compared between survival and non-survival groups. Univariate logistic regression and multivariate analysis were done to determine the association between survival and IL-6 reduction at 24 h after ICU admission.

**Results:**

A total of 123 patients were enrolled. The concentration (in pg/mL) of IL-6 at T_o_ was 413.3 in survivors and 530.0 in non- survivors. At T_24_, the IL-6 level was 65.4 for survivors and 286.9 for non-survivors. The survival rate was 39.0%. At T_24_, the concentrations of IL-6 and the reduction in IL-6 level were predictors of survival in patients with sepsis and septic shock. We found a significant association between IL-6 reduction and survival at ≥86% with Odds Ratio (OR) 5.67, 95% Confidence Interval (CI); 1.27–25.3, compared with an increase in the IL-6 rate of change.

**Conclusions:**

Our findings suggested that a reduction in the IL-6 level of ≥86% at 24 h from ICU admission is a survival predictor for patients with sepsis and septic shock in our population.

## Background

Sepsis and septic shock are common problems in intensive care units (ICUs). The corresponding mortality for sepsis and septic shock is 24.3–60.0% even in developed countries [1–5]. This mortality rate has decreased 1.3% annually since 2012 due to early diagnosis and appropriate empiric antibiotic therapy [6, 7]. However, the treatment of sepsis and septic shock remains expensive. The total hospital cost for patients with severe sepsis in the USA increased from $15.4 billion in 2003 to $24.3 billion in 2007 [8].

Monitoring of the severity of sepsis or septic shock is a considerable challenge. Most decisions about patients with sepsis and septic shock are based on clinical and laboratory findings that have limited accuracy. Biomarkers such as cytokines have been suggested to be predictors of severity and mortality in patients with sepsis and septic shock in several studies [7, 9, 10]. Acute Physiology and Chronic Health Evaluation (APACHE) II and Sequential Organ Failure Assessment (SOFA) scores have been used widely as predictors of severity in sepsis and septic shock [11].

The cytokine interleukin (IL)-6 is essential in cell development, initiation of innate immunity and cell functions in adaptive immunity [12]. In 2016, Klag T et al. studied 20 severe bacterial sepsis patients and showed that a rapid decline in IL-6 concentration after 24–48 h to or below baseline value was evidence of successful empiric antibiotic therapy, and was a survival predictor [13]. Juan et al. in Argentina showed that the IL-6 level decreased after 72 h from admission in 48 sepsis or septic shock patients, and was a survival predictor. They showed that the IL-6 at admission was 161 ± 24 pg/ml in survivors and 121 ± 17 pg/ml at 72 h after admission (*p* = 0.04) [14]. How changes in plasma levels of cytokines may be used to predict survival in patients with sepsis and septic shock for a Vietnamese population is not known. We determined to find out if reductions in the plasma concentrations of the cytokines tumor necrosis factor (TNF)-α, IL-6, and IL-10, and the rate of change of IL-6 at 24 h after ICU admission were survival predictors for patients with sepsis and septic shock at an ICU in Ho Chi Minh City, Vietnam.

## Methods

We included patients admitted to the ICU at Cho Ray Hospital (Ho Chi Minh City, Vietnam) prospectively from October 2014 to October 2016. The ICU has 36 beds amongst a total of 2600 hospital beds. It cares for surgical, medical and post-operative patients. Patients who had undergone surgical procedures for severe head injury or who had open-heart surgery were excluded. There are 12 physicians and 36 nurses working in a shift system in the ICU.

Patients were eligible for inclusion if they fulfilled the criteria for the diagnosis of sepsis and septic shock defined by the American College of Chest Physicians/Society of Critical Care Medicine Consensus Conference in 2001 [15]. The doctors in charge explained to patients’ relatives the research. If they agreed to be enrolled, they signed consent.

Clinical data were collected using pre-designed forms. Patients were followed up for the duration of their ICU stay and their duration of hospital stay, and their survival rate was ascertained.

Blood samples were collected at the time of ICU admission (T_0_) and at 24 h after ICU admission (T_24_). The 3 ml venous blood sample was taken and then centrifuged to separate the serum, which was stored at − 20 °C for further use. The concentrations of TNF-α, IL-6 and IL-10 were measured using an enzyme-linked immunosorbent assay. The Human IL-6 ELISA (Enzyme-Linked Immunosorbent Assay) kit was an in vitro enzyme-linked immunosorbent assay for the quantitative measurement of human IL-6 in plasma. The IL-6 enzyme-linked immunosorbent assay kit was purchased from Biochip Randox (County Antrim, United Kingdom). The blood cell analyzer was purchased from Randox company. The IL-6 rate of change at 24 h after ICU admission was measured using the following equation: IL-6 rate of change at 24 h from ICU admission = (IL-6 level at T_0_ − IL-6 level at T_24_)/IL-6 level at T_0_ × 100%.

The therapy for sepsis and septic shock was according to the SSC 2012 guidelines. Empiric antibiotic therapy was used according to local Hospital Antibiotic Guidelines. Renal replacement therapy was indicated in sepsis or septic shock with acute kidney injury, but no cytokine blood purification was used.

The Student’s *t*-test and Mann–Whitney test were used to determine the significance of difference between survival and non-survival groups. The univariate logistic regression and multivariate regression analyses were done to identify which variables were associated with survival. We also divided the IL-6 reduction into quartiles. We adjusted for the variables with the *p*-value over 0.1 for multivariate logistic regression such as BUN, Creatinine, aPTT, pH, HCO_3_^−^, gender and age.

## Results

One hundred and twenty-three patients were enrolled. Table 1 shows the characteristics of those patients who survived and those who died. Patients diagnosed with sepsis and septic shock admitted to the ICU who subsequently died had high APACHE II and SOFA scores. APACHE II score, SOFA score and the number of dysfunctional organs were significantly different between non-survival and survival groups (*p* < 0.05). The duration of ICU stay for survivors was longer (median 18, the interquartile range (IQR) 13–31 days) than that of non-survivors (7, IQR 3–16). Also, 98/123 (79.7%) of cases had mechanical ventilation, and the median number of days of mechanical ventilation was 5 (IQR 3–12) days. The overall survival rate was 39.0%.

**Table 1:** Characteristics of patients upon ICU admission and the mortality rate (*n* = 123)

Variable	Survival (*n* = 48)	Non-survival (*n* = 75)	*p*
Age (years), (median, IQR)	62 (46–75)	54 (43–73)	0.18**
Pre-ICU stay (days),(mean ± SD)	3.8 ± 5.7	2.9 ± 4.9	0.11**
Duration of hospitalization (days) (mean ± SD)	21.5 ± 13.8	12.7 ± 15.4	< 0.01*
Duration of ICU stay (day) (median, IQR)	18 (13–31)	7 (3–16)	0.03**
APACHE II score (mean ± SD)	18.0 ± 5.8	26.6 ± 7.9	< 0.01*
SOFA score (mean ± SD)	9.1 ± 2.9	11.6 ± 3.6	< 0.01*
Mechanical Ventilation (n, %)	32/48 (66.7)	66/75 (88.0)	< 0.01*
Days of ventilation (median, IQR)	5 (3–12)	4(1–15)	0.15**
Sites of infection	Prevalence	Mortality	
Gastrointestinal tract	56.1%	66.7%	
Respiratory tract	21.1%	61.5%	
Urinary tract	7.3%	44.4%	
Others	15.4%	47.2%	

(*) Student’s *t*-test, (**) Mann–Whitney test, IQR: interquartile range, SD: standard deviation

Table 2 shows the laboratory parameters at ICU admission and IL-6 rate of change at 24 h after ICU admission. Levels of blood–urea–nitrogen (BUN) and activated partial thromboplastin time (aPTT) were higher for non-survivors (*p* = 0.02). Levels of arterial pH and bicarbonate among non-survivors were significantly lower than those of survivors (*p* = 0.04 and 0.01 respectively). The rate of change in IL-6 level at T_24_ was significantly different between survival and non-survival groups (p = 0.04).

**Table 2:** Laboratory parameters upon ICU admission and IL-6 rate of change after 24 h ICU admission (*n* = 123)

Laboratory parameters	Survival (*n* = 48) Mean ± SD	Non- survival (*n* = 75) Mean ± SD	p (Student’s *t*-test)
Hemoglobin (g/dL)	11.3 ± 2.3	11.0 ± 2.6	0.50
White blood cells (K/mm^3^)	20.6 ± 14.2	18.1 ± 13.2	0.34
Platelets (K/mm^3^)	164 ± 126	193 ± 134	0.24
Blood glucose (mg/dL)	129.3 ± 80.8	138.6 ± 77.8	0.55
Blood–urea–nitrogen (mg/dL)	35.7 ± 21.6	45.9 ± 25.1	0.02
Creatinine (mg/dL)	1.97 ± 1.5	2.47 ± 1.6	0.08
Bilirubin (mg/dL)	2.40 ± 2.4	3.34 ± 4.6	0.17
Prothrombin time (s)	16.9 ± 5.4	18.6 ± 10.0	0.27
aPTT (s)	35.9 ± 9.4	42.9 ± 20.8	0.02
pH	7.33 ± 0.1	7.28 ± 0.10	0.04
PaCO_2_ (mmHg)	35.6 ± 7.8	33.5 ± 9.3	0.21
PaO_2_ (mmHg)	120.4 ± 79.3	129.9 ± 99.6	0.58
HCO^−^_3_ (mmol/L)	19.0 ± 4.4	16.4 ± 5.8	0.01
C-reactive protein (mg/L)	123.3 ± 45.7	122.4 ± 60.8	0.93
Procalcitonin (ng/dL)	42.5 ± 109.8	54.4 ± 91.4	0.16
Lactate (mmol/L)	4.2 ± 2.6	5.3 ± 4.3	0.59
IL-6 rate of change	0.8 ± 4.5	−0.7 ± 0.3	0.04

aPTT, activated partial thromboplastin time; PaCO_2_, arterial pressure of carbon dioxide; PaO_2_, arterial pressure of oxygen; HCO^−^_3_, bicarbonate

IL-6 rate of change = (IL-6 (T_0_) – IL-6 T_24_)/ IL-6 T_0_

**Fig. 1:**
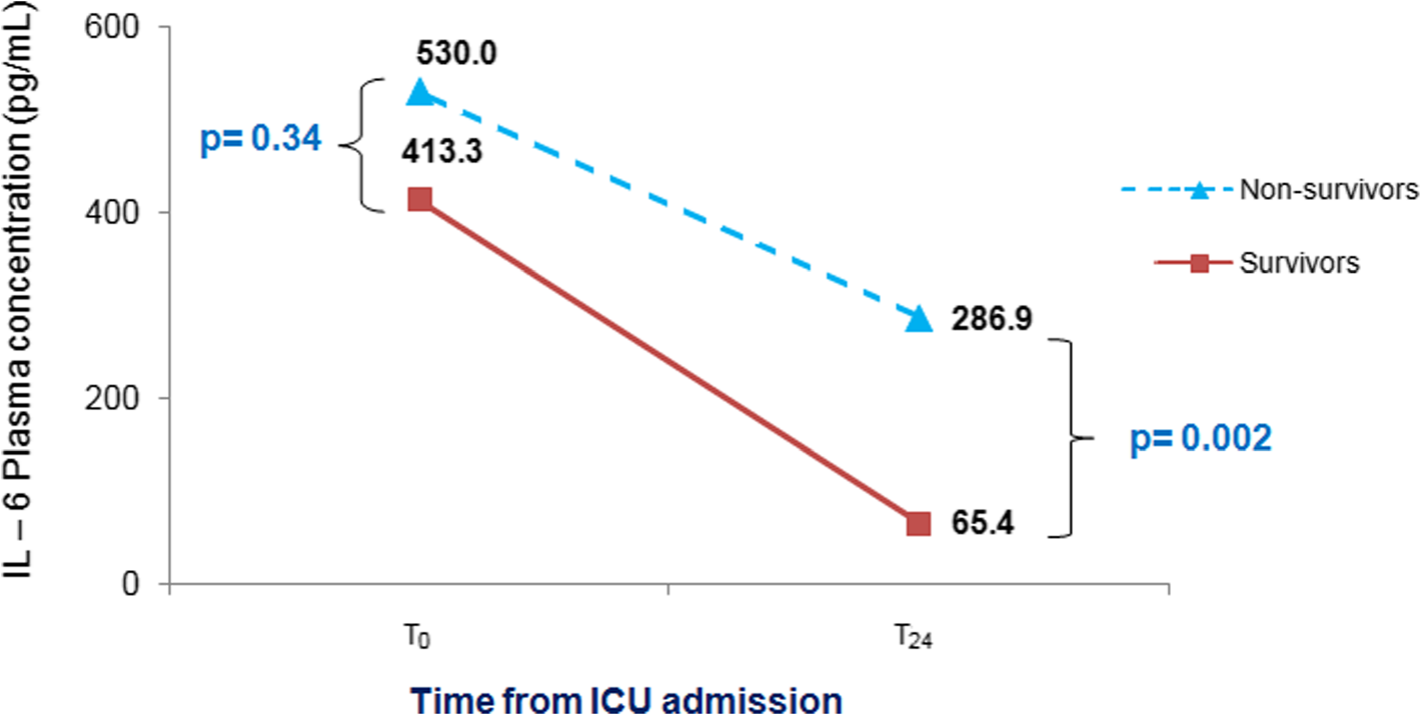
The IL-6 change after 24 h of ICU admission

Figure 1 shows that the concentration of IL-6 was similar at T_0_ in both groups (*p* = 0.34), but was significantly reduced at T_24_ in the survival group compared to the non-survival group (*p* = 0.002).

Table 3 shows cytokine concentrations at T_0_ and T_24_ in survivors and non-survivors. There were significant differences in the levels of IL-6 (*p* < 0.01) and IL-10 (p < 0.01), and in the IL-6 rate of change at 24 h after ICU admission (*p* = 0.03).

**Table 3:** Association of IL-6 reduction rate and survival for sepsis and septic shock (*n* = 123)

	Univariate		Multivariate	
	OR	(95%CI)	OR	(95%CI)
IL-6 reduction rate (quartile)				
Q1: 100% to 86%	5.08	(1.60–16.1)	5.67	(1.27–25.3)
Q2: 85% to – 50%	1.45	(0.52–4.11)	1.86	(0.44–7.94)
Q3: 0.49% to 0%	0.64	(0.22–1.83)	0.54	(0.14–2.02)
Q4: increased	ref		ref	

Adjusted for age, sex, BUN, Cre, aPTT, pH, HCO_3_^−^

ref: Reference

*OR* Odds Ratio, *CI* Confidence Interval

IL-6 reduction rate = (IL-6 (T_0_) – IL-6 T_24_)/ IL-6 T_0_

T_0_: At ICU admission time

*T*_*24*_: 24 h after ICU admission

Table 4 shows the association of IL-6 reduction rate and survival for sepsis and septic shock with logistic regression analysis. The quartiles for IL-6 reduction rate were 100% to 86%, 85% to 50%, 49% to 0%, and increased from 0%. We found a significant association between IL-6 reduction and survival at ≥86% reduction, with an Odds Ratio (OR) 5.67, 95% Confidence Interval (CI); 1.27–25.3, compared with the increase in the IL-6 rate of change. According to logistic regression, the rate of change of TNF-α and of IL-10 were not a predictor of survival.

**Table 4:** Association of IL-6 reduction rate and survival for sepsis and septic shock in logistic regression analysis (*n* = 123)

	Univariate		Multivariate	
	OR	(95%CI)	OR	(95%CI)
IL-6 reduction rate (quartile)				
Q1: 100% to 86%	5.08	(1.60–16.1)	5.67	(1.27–25.3)
Q2: 85% to 50%	1.45	(0.52–4.11)	1.86	(0.44–7.94)
Q3: 49% to 0%	0.64	(0.22–1.83)	0.54	(0.14–2.02)
Q4: Increased	ref		ref	

Adjusted for age, sex, BUN, Cre, aPTT, pH, HCO_3_^−^

ref: Reference

*OR* Odds Ratio, *CI* Confidence Interval

IL-6 reduction rate = (IL-6 (T_0_) – IL-6 T_24_)/ IL-6 T_0_

T_0_: At admission time

*T*_*24:*_ 24 h after ICU admission

## Discussion

We aimed to investigate if reductions in the plasma concentrations of TNF-α, interleukin (IL)-6 and IL-10, and of the IL-6 rate of change at 24 h after ICU admission were survival predictors for Vietnamese patients with sepsis and septic shock. The results showed that IL-6 reduction at the level of ≥86% at 24 h from ICU admission could be a survival predictor for our patients with sepsis and septic shock. Our study showed that patients who had decreased IL-6 level after 24 h of ICU admission at ≥86% were 5.68 times more likely to survive compared to non-survivors.

IL-6 plays a key part in the systemic inflammatory response. Increased IL-6 levels in plasma have been identified in severe forms of sepsis, and correlate with an increased prevalence of mortality [4]. Levels of IL-6 and CRP were shown to be unreliable predictors for 102 critically ill patients admitted to the ICU of a tertiary hospital in Serbia [16]. In healthy adults without an ongoing inflammatory process, the IL-6 concentration ranged from 0.2 to 7.8 pg/mL, whereas the IL-6 concentration in adults with sepsis could be > 1600 pg/mL [17]. The IL-6 level is also proven as a predictor of acute kidney injury [18].

Reduction in IL-6 level denoted the patients’ response to treatment. This finding also implies that the initial empiric antibiotic therapy was appropriate and should continue until formal culture results. Besides that, any treatment that can reduce IL-6 concentration would be supportive for these patients. Mohammad and co-workers found that, compared with the IL-6 level upon ICU admission, the IL-6 concentration at ICU discharge predicted all-cause mortality [19]. A meta-analysis in 2010 by Jaffer and colleagues, using data from human studies and experimental animal models, suggested that pro- and anti-inflammatory cytokines are released following a variety of initiating stimuli (e.g., endotoxin release, complement activation, ischemia–reperfusion injury). “Cytokine adsorption therapy” provides a potential solution to improving outcomes following systemic inflammatory response syndrome [20]. A study by Marissa and co-workers in 2010 showed that, 24 h after ICU admission, cytokine concentrations in low- and high-concentration subgroups were significantly different in the survival and non-survival groups [21]. The high cytokine concentrations 24 h after ICU admission were predictors of mortality for sepsis patients [21]. IL-6 clearance by extracorporeal membrane oxygenation and continuous renal replacement therapy has been shown to lower the mortality [22].

Sepsis and septic shock are the most common causes of death in the ICU [23, 24]. In recent years, the age of patients suffering from sepsis admitted to the ICU has been increasing [5]. The mean age of our study cohort was 58.2 ± 18.8 years, the duration of ICU stay was 6 (IQR 3–12) days, and 39.0% of patients survived. Compared with the previous studies at Cho Ray Hospital undertaken in 2013 and 2014, the mean age of our study cohort was higher and more patients died [25, 26]. These observations could have been because our patients had more severe disease, were resuscitated later, and or discharged earlier from the ICU compared with the studies carried out in 2013 and 2014. Also, Cho Ray Hospital has a post-resuscitation area where recovering ICU patients can be cared for. The prevalence of mortality was higher than that observed in other studies because our patients underwent treatment in other departments before ICU admission. Also, the APACHE II and SOFA scores upon ICU admission were high indicating severe illness.

Several cytokines could be indicators for severity and mortality for patients with sepsis and septic shock. In 2016, Stalder and colleagues evaluated 129 sepsis patients. They recorded an in-hospital mortality of 26%, and showed that plasma levels of growth arrest-specific gene 6 within 24 h of ICU admission could predict mortality for sepsis patients [27].

In our analysis, IL-10 concentration at T_24_ was also significantly different. However, the IL-10 rate of change was not calculated as IL-10 is a pre - inflammatory biomarker, and is used to diagnose sepsis rather than survival prognosis in sepsis patients [28]. Also, IL-10 is not tested as a point of care test, and is not widely used clinically.

Whether pro-inflammatory biomarkers such as C-reactive protein (CRP) or procalcitonin could be predictors for mortality remains controversial [29]. Biron and co-workers showed that a single procalcitonin concentration is not a good predictor for sepsis and septic shock, but that serial procalcitonin concentrations with high clearance of procalcitonin were reliable predictors [29]. However, a meta-analysis involving 4467 patients showed that procalcitonin-guided therapy did not lower the mortality [30].

We observed a significant increase in mean plasma levels of CRP (122.7 mg/L) and procalcitonin (49.8 ng/mL), but a significant difference between the survival group and non-survival group was not observed (*p* = 0.931 and 0.159, respectively). Levels of CRP and procalcitonin were thus poor predictors for survival. Other studies undertaken at Cho Ray hospital have elicited similar results [25, 26]. Measurement of the concentrations of CRP and procalcitonin thus aids the diagnosis of infection, but has low accuracy for predicting survival.

In this research, our sepsis and septic shock patients were treated according to Surviving Sepsis Campaign 2012 guidelines. The empiric antibiotic therapy was based on our hospital antibiotic guideline. The research was finished before the Surviving Sepsis Campaign guidelines 2016 were published [31].

### Limitations

Our study had two main limitations. First, the time to sepsis onset could not be identified accurately. In this study, we did not find how long our patients may have been affected by sepsis before their admission. Further research may be needed with more frequent monitoring of vital signs to detect early sepsis based on the new guidelines SSC 2016. Second, this research was performed at Cho Ray hospital, a tertiary teaching hospital where modern medical equipment and techniques are available. The generalizability of this finding for all Vietnamese hospitals may be limited. However, there were several hospitals which have similar levels of equipment and facilities in Vietnam, who could apply this research.

## Conclusions

Our findings indicate that a reduction in IL-6 level of ≥86% at 24 h from ICU admission was a survival predictor for sepsis and septic shock patients comparing with patients who had an increasing IL-6 rate of change. This finding also supports continuing the initial empiric antibiotic therapy in sepsis and septic shock patients. Hence, in terms of clinical applications, measurement of the IL-6 level should be done upon admission, and at 24 h after ICU admission in sepsis and septic shock patients.

## Abbreviations

APACHE: Acute Physiology and Chronic Health Evaluation
AUC: Area under the curve
BUN: Blood-urea-nitrogen
CRP: C-reactive protein
ICU: Intensive care unit
IL: Interleukin
IQR: Interquartile range
SOFA: Sequential Organ Failure Assessment
SSC: Surviving Sepsis Campaign
TNF: Tumor necrosis factor
